# Complex problems and unchallenged solutions: Bringing ecosystem governance to the forefront of the UN sustainable development goals

**DOI:** 10.1007/s13280-017-0918-6

**Published:** 2017-04-22

**Authors:** Liette Vasseur, Darwin Horning, Mary Thornbush, Emmanuelle Cohen-Shacham, Angela Andrade, Ed Barrow, Steve R. Edwards, Piet Wit, Mike Jones

**Affiliations:** 1 Department of Biological Sciences, UNESCO Chair in Community Sustainability: From Local To Global, Brock University, 1812 Sir Isaac Brock Way, St Catharines, ON L2S3A1 Canada; 2 Commission on Ecosystem Management, International Union for the Conservation of Nature, 28 Rue Mauverney, 1196 Gland, Switzerland; 3 School of Environmental Planning, University of Northern British Columbia, Prince George, BC Canada; 4 Department of Zoology, Tel Aviv University, 69978 Tel Aviv, Israel; 5 Conservation International-Colombia, Carrera 13 No. 71-41, Bogotá, Colombia; 6 Global Ecosystem Management Programme, International Union for the Conservation of Nature, 28 Rue Mauverney, 1196 Gland, Switzerland; 7 Swedish Biodiversity Centre, Almas allé 8, 750 07 Uppsala, Sweden; 8 Department of Biological Sciences, Brock University, 1812 Sir Isaac Brock Way, St Catharines, ON L2S 3A1 Canada; 9 Sustainable Communities International, 14748 Goodrich Creek Lane, Baker City, OR 97814 USA

**Keywords:** Conservation, Ecosystems, Environmental degradation, Governance, International conventions, Sustainability

## Abstract

Sustainable development aims at addressing economic, social, and environmental concerns, but the current lack of responsive environmental governance hinders progress. Short-term economic development has led to limited actions, unsustainable resource management, and degraded ecosystems. The UN Sustainable Development Goals (SDGs) may continue to fall short of achieving significant progress without a better understanding of how ecosystems contribute to achieving sustainability for all people. Ecosystem governance is an approach that integrates the social and ecological components for improved sustainability and includes principles such as adaptive ecosystem co-management, subsidiarity, and telecoupling framework, as well as principles of democracy and accountability. We explain the importance of ecosystem governance in achieving the SDGs, and suggest some ways to ensure that ecosystem services are meaningfully considered. This paper reflects on how integration of these approaches into policies can enhance the current agenda of sustainability.

## Introduction

Without ecosystems, human life is not possible on this planet. This seems to be a simple and obvious statement but all too often it is not taken seriously. Humans continue to forestall taking action to maintain the integrity of ecosystems, secure in the belief that technology and innovation will fix all problems (Keulartz 2012). Based on such ideology, continuous population and economic growths with free markets and globalization is believed to be the only way to live and develop, and this seems politically acceptable even if we admit that we live in a finite world. Recent evidence, however, indicates otherwise (MEA 2005; OECD 2008; Liu et al. 2015). Since humans have evolved into societies, they have increasingly impacted ecosystems worldwide, believing they “control” nature. Industrialization, population growth, and rising consumption have brought new levels of impacts and unforeseen environmental consequences since the Industrial Revolution.

With recent advances in our understanding of the interdependence between social and ecological systems and how environmental and climate changes further complicate any simple linear approaches, the emerging paradigm in resource management is beginning to recognize the “wicked” nature of ecosystem governance (Levin et al. 2012). Wicked problems are ones that “defy complete definition and easy or complete solutions due to the inherent and constantly evolving complexity of the system at stake” (Moser et al. 2012, p. 52). Here, we refer to “wicked” as any issue that relates to ecosystem survival (including human) that cannot be solved through the application of deterministic science. Wicked problems relate to the complexity of ecosystems and the plurality of human perspectives on the definition of environmental problems and solutions. Unlike simple engineering problems, they require new approaches that respect the interplay between social and ecological components of the system and the non-linear nature of our world (Cosens 2013). Within the Anthropocene epoch (Lewis and Maslin 2015), human impacts on ecosystems have now reached what some have called the ‘planetary boundary’ (Folke et al. 2011; Steffen et al. 2011a; Sachs 2012; Griggs et al. 2013). Current human demands on ecosystem services are such that we may be close to dangerous thresholds and tipping points, with some of these services already threatened and others approaching irreversible degradation (Foley 2010; Steffen et al. 2011b; Wijkman and Rockström 2012). Without respecting ecosystems that, for instance, provide food and water, allow for purification of the air and water, or moderate extreme events, there is little hope of preventing our environmental systems from exceeding immutable planetary boundaries.

The international community has tried to find ways to reduce the probability of exceeding the planetary boundaries. The Millennium Development Goals (MDGs 2000) were formulated from the desire by most countries to improve human life in a sustainable manner. The least developed countries (LDCs) were the main focus with the hope that the MDGs would lead to better integration with the developed world economy. Countries and organizations were encouraged, initially high buy-in, to help LDCs move forward with the MDGs. Fifteen years later, we know that, while some countries have made substantial progress towards poverty alleviation, gender equality, etc., such goals still remain out of reach for many others (Clemens et al. 2007; United Nations 2014). There are several reasons for this limited success, including the failure of most developed countries to honor their development assistance promises, political greed or unrest, lack of adequate accounting and monitoring of initiatives, population growth absorbing any progress made, etc. Current life styles, technological advancements, economic drivers of over-consumption, and rapid globalization have all contributed to exacerbating the inadequacy of the system to respond to basic human needs. Compounding these challenges are the continued and escalating impacts associated with climate change and environmental degradation (Mearns and Norton 2010). These wicked problems challenge the capacity of all nations, rich and poor, in achieving any significant progress towards sustainability that encompasses ecological, social, and cultural systems.

**Fig. 1:**
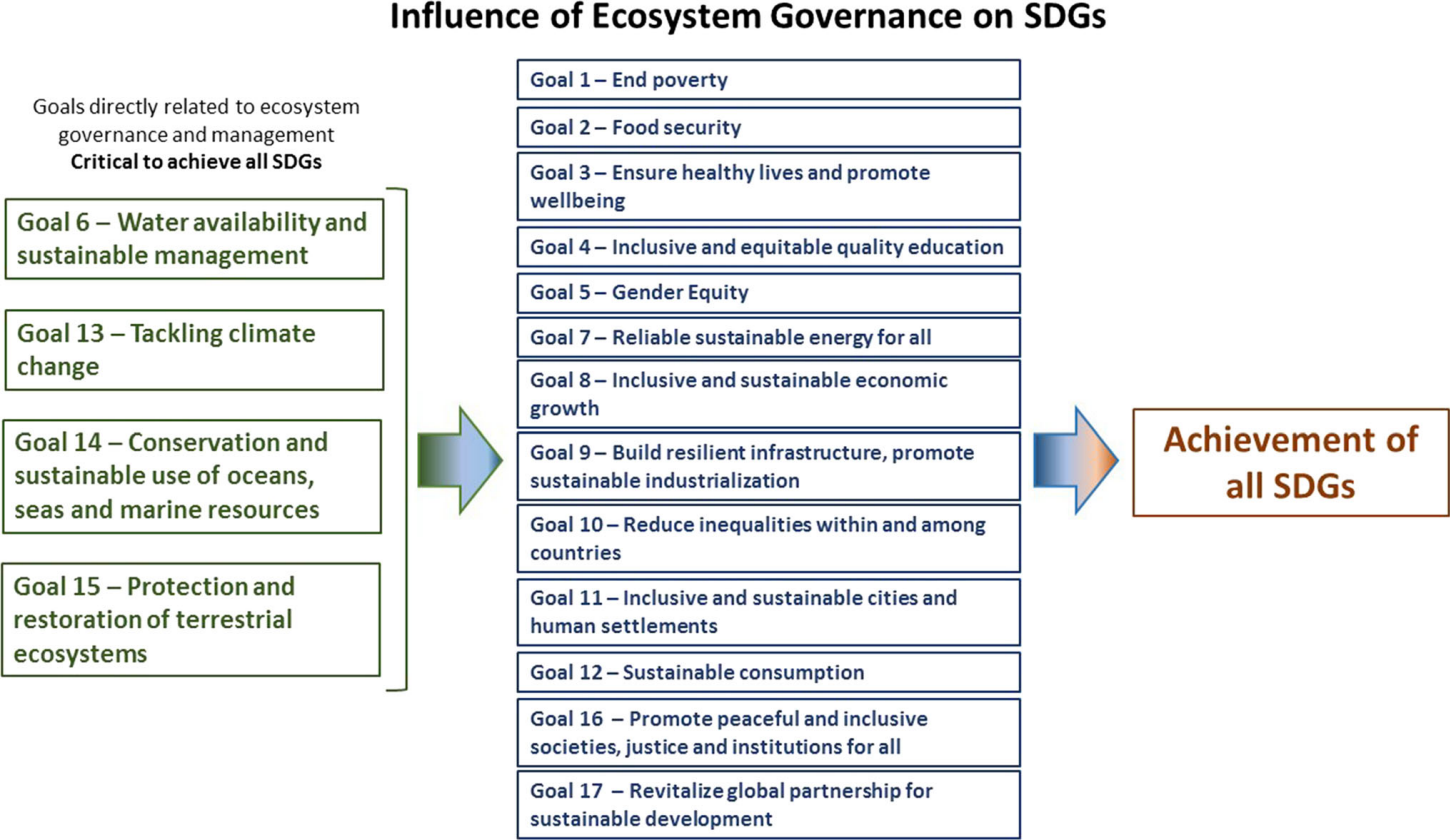
Direct and indirect influence of ecosystem governance on the achievements of SDGs

Sustainable development goals, whether the MDGs or the 2015 UN Sustainable Development Goals (SDGs), are based on the early analogy of sustainability, where sustainability depends on three pillars: economic, social, and environmental—each of equal importance. In the MDGs, the human and development components were profiled over the environmental pillar. Yet, environmental degradation has been a major barrier to achieve MDGs (Sachs et al. 2009). The current culture of consumerism that is often promoted in development favors land use practices that can undermine the capacity of an ecosystem to maintain its functions and disregards the waste streams (e.g., pharmaceuticals) of consumerisms and their effects on ecosystem health. There is an increased recognition of the importance of the environment in the SDGs in terms of climate change and sustainable use of natural resources. So, where do these “environmental goals” now stand? It appears that we have learned little from the MDG process, as the environment not only requires a higher profile but also needs to be integrated across the SDGs. The International Council for Science (ICSU 2015) reports that despite major improvements from the MDGs, the SDGs would be improved if they were based on more recent scientific evidence, with more measurable and time-sensitive targets, and were better integrated (Liu et al. 2015; Fig. 1). We support these statements and add that the condition of a nation’s ecosystems and the way it manages them directly affect the nation’s ability to address the majority of the SDGs.

The current assumption that ecosystems are free, static, permanent, and can “take care of themselves” when impacted or disturbed, and therefore require little attention, is inaccurate, untrue, and misses the fundamental reality that all 17 SDGs rely on resilient and diverse ecosystems. Ecosystems are, therefore, not merely part of one leg of the stool, but the platform on which the stool stands or the foundational layer on which society survives (Griggs et al. 2013). If humanity does not consider the impacts on ecosystems nor finds more equitable solutions for their conservation and management, there is little doubt that the three-legged stool will be reduced to two legs (or pillars) and collapse. This is where the notions of ecosystem governance, and its direct link to the ecosystem approach and the use of nature-based solutions to provide sustainable ecosystem services, should be a cornerstone and a priority approach to achieve the SDGs. In this perspective paper, we underline the importance of ecosystem governance in achieving the SDGs, and explore ways to ensure that ecosystem services and biodiversity are seriously considered in all future actions, as they represent a nation’s natural capital and heritage. Ecosystem governance, later defined in the paper, is an approach that enhances the connection between ecosystems and society through increased fairness and balance between the needs and limitations of what is provided by ecosystems. We propose that ecosystem services and governance are central in planning and decision making if we are to curb further losses of biodiversity and enhance social-ecological resilience as we aspire towards a sustainable future.

## Ecosystem governance: Building on the main international environmental conventions

For a long time, it was wrongly believed that poverty was the major and root cause of environmental degradation and that by reducing poverty through programs like the MDGs, environmental issues would lessen (Adams et al. 2004; Pearce 2011). While poverty may contribute to environmental degradation, issues such as land use conversion and degradation, invasive species, and the overexploitation of natural and mineral resources are more serious and insidious (Adams et al. 2004). Overexploitation occurs where corruption, lobbying of some corporations and organizations, and weak environmental policies (or lack of enforcement) enable exploitation of the environment and local communities. It can lead to the implementation of perverse policies, often in the name of economic development (Palmer and Di Falco 2012).

Since the 1970s, to resolve some of these issues, the world’s nations have developed and implemented conventions and targets to move towards a more sustainable future. Beside the MDGs, other major global environmental conventions have been ratified to support the goals of ecosystem and biodiversity conservation such as the Convention on Biological Diversity (CBD), the United Nations Framework Convention on Climate Change (UNFCCC), the Convention on International Trade in Endangered Species of Wild Fauna and Flora (CITES), and the Ramsar Convention on Wetlands of International Importance. In addition, more specific conventions have been developed such as the United Nations Convention to Combat Desertification (UNCCD), which was adopted to respond to critical changes happening to a particular biome across the planet. Are these conventions and targets an indication of our failure to address any meaningful integration of ecosystems and the services that they provide into our decision-making processes? In reality, what is needed is the social acknowledgement of our interdependence with the world’s ecosystems in all non-environmental agreements and bodies such as the World Trade Organization (WTO). WTO promotes global trade but this comes at the expense of the environment such as increasing CO_2_ emissions and spreading of invasive species, issues not currently addressed by the organization (Liu et al. 2015). Our continued failure to recognize the importance of the environmental pillar appears to be addressed through “band aid repairs” in the form of new and revised conventions, without addressing the human-nature nexuses (Liu et al. 2015).

The short-term view of government economic priority setting and elected politicians generally clash with the long-term view of the SDGs. As Bosselman et al. (2008, p. 11) argue, “governments are elected by the citizens, that is, by us. Only insofar as civil society supports and reaffirms the idea of economic growth, can we blame governments for missing the point of sustainability.” For this challenge, they propose some solutions such as having political tenure lasting for 10 years but with having a tenth of them elected annually to avoid power accumulation or the implementation of coercive legislative framework to ensure all governmental actions have sustainability integrated. Having a greater role of public participation into decisions through advisory committees (e.g., Joint Public Advisory Committee of the North American Commission for Environmental Cooperation) may also help change the views of people regarding sustainability, as this will help foster greater accountability and environmental democracy.

## Ecosystem in ecosystem governance

Ecosystems are composed of living and non-living elements interacting together and are the basic structure on which all life on Earth is based. All ecosystems are mediated by societal and cultural actions at multiple scales. Humans with their economic activities, cultures, and religious beliefs have modified ecosystems and altered over 60% of ecosystems (Diaz 2006) and many elements are either degraded or used unsustainably. Ecosystem services are defined as the essential elements that all species, including humans, depend upon for their survival (Vasseur et al. 2002a) or as the benefits people obtain from ecosystems (Costanza et al. 1997; MEA 2005) including provision (e.g., water, food, or raw material); maintenance and regulation (e.g., pollination, water purification, climate regulation, or erosion prevention); and cultural (e.g., recreational, educational, cultural, or spiritual services). Depending on the framework, a fourth category, supporting services (e.g., nutrient cycling, primary production) are considered to include the services necessary for the production of all three of ecosystem services categories (MEA 2005). These sometimes referred to as habitat services help highlight the importance of ecosystems to the provision of habitat for migratory species (De Groot et al. 2002, TEEB 2010). In another widely used classifications—e.g., CICES, the Common International Classification of Ecosystem Services (see Haines-Young and Potschin 2013), and IPBES, the Intergovernmental Science-Policy Platform on Biodiversity and Ecosystem Services (see Díaz et al. 2015), this category is integrated into the maintenance and regulation category. Ecosystem services represent the backbone of all current and potential economic, social, and cultural growths in any community—rural and urban. Maes et al. (2014, p. 15) state that research should investigate “the multifunctionality of ecosystems for sustaining long-term human wellbeing.” The long-term, sustained provision of ecosystem services is therefore the definitive goal.

The ecosystem services framework highlights the dependency of human wellbeing on ecosystems and their benefits (Fisher et al. 2009). This framework helps demonstrate how the disappearance of biodiversity and services due to anthropogenic environmental and climate changes has direct effects on ecosystem functionality, which underpins critical services for human wellbeing (Braat and De Groot 2012). However, ecosystems or values are difficult to assess as most of them are outside the markets, despite their non-tractable benefits (de Groot et al. 2012). A common criticism of the concept of ecosystem services is that its anthropocentric focus excludes the idea of ecosystems and biodiversity as inherently valuable beyond human needs (Deliège and Neuteleers 2014; Schröter et al. 2014) although it is more recently being acknowledged in the IPBES framework. The other challenge is that most current management practices and policies use a reductionist approach where only one component (e.g., water, land, or food) is addressed at a time, despite the interconnection at the system level (Liu et al. 2015).

Recognizing that we are dealing with wicked problems is the first step to solving the complexity of the situation. Applying the subsidiarity principle and allowing people to develop multiple solutions to problems reduce the emphasis on their “wickedness”. The subsidiarity principle refers to the importance of the contribution of individuals in communities and requires that decisions and actions are done and are accountable at the lowest appropriate governance level (Vischer 2001; Martinez de Anguita et al. 2013). If well thought, subsidiarity should lead to “empowerment of individuals acting together through social groupings and associations” (Vischer 2001, pp. 109–110) and emphasizes the importance of common good. It brings greater social awareness and the importance for people to understand their impacts on their own systems. Vischer (2001, p. 117) states that: “From a subsidiarity perspective, these attributes are invaluable because they instill a sense of responsibility for one’s self and one’s surroundings, along with the tools needed to act in betterment of both.” Thus, solutions can be more socially acceptable and locally adaptive. Subsidiarity, however, does not mean devolution of all powers as the state remains important to ensure that minimum standards are respected and to mediate against various issues that can occur within the state or internationally (Vischer 2001).

Under the subsidiarity principle, solutions can use a more holistic social-ecological system (SES) view, where human actions, including cultural systems, must be mitigated and adapted to reduce negative consequences on the ecological systems (Folke et al. 2005; Dickman et al. 2015). Under a social-ecological approach, communities would have to ensure protection of ecosystems to continue providing life-sustaining services in the form of clean air, potable water, fertile soils, and natural products, as part of the common good. Managing human activities in a more local and sustainable manner can result in a greater understanding of integrated and adaptive ecosystem management principles and practices.

This would also mean that governments should play a greater mediating role, for example, by enhancing the capacity of their ministries to assist communities in more sustainable actions, on a temporary basis and in a limited and empowering manner. Assistance would be needed in cases where socio-economic conditions or extreme circumstances lead communities to not be able to manage (Vischer 2001). For Carozza (2003), subsidiarity also relates to the need for higher level of government to find effective and equitable solutions to address global-scale challenges through the issue of human rights. As ecosystems are all interconnected, especially with globalization and the virtual market, governments will have a crucial role to play in ensuring that a human-nature nexus framework recognizes the spatial and temporal interdependency of ecosystems is respected (Liu et al. 2015). In addition, as governmental agencies usually have relatively narrow mandates (e.g., Ministries of Environment vs. Natural Resources) with limited cooperation among them, governments will have to promote greater integration between the different ministries so that social-ecological considerations are fully included in any decisions (Karkkainen 2002). If governments acknowledge the intricate linkages between the SES and devolved governance to the lowest accountable level, more effective steps towards solving wicked problems and sustainability could be defined. Vischer (2001, p. 120) argues “subsidiarity is not simply an abstract principle of governance, but rather a practical framework for solving real problems”. Interestingly, even the Constitution of the United States of America was influenced by the subsidiarity principle, although in the past century the country has deviated from the principle (Vischer 2001).

Ecosystem governance is defined by the International Union for Conservation of Nature (IUCN) as “the interactions among structures, processes, and traditions that determine how power and responsibilities are exercised, how decisions are taken, and how citizens or other stakeholders have their say in the management of natural resources—including biodiversity conservation” (IUCN 2004). This definition integrates the notion of SES and subsidiarity principle (also referred to as environmental subsidiarity; Martınez de Anguita et al. 2013), where communities play a role in using various ecosystem services as well as defining solutions and sustainable development pathways, while being the stewards of those ecosystems. This also acknowledges the importance of connections between people and ecosystems, recognizing that without sustainable healthy ecosystems there can be no sustainable healthy communities (Vasseur et al. 2002a).

The challenge now involves addressing the wicked problems, such as climate change and land use conversion and degradation, using an ecosystem governance approach to reduce current and future vulnerabilities (Pahl-Wostl et al. 2012). Much of the attention on governance of ecosystems or a part of them (e.g., water management) over the past several years has focused on the influence that human (social) interventions have had on ecological systems or, alternatively, on the effects of rapidly changing environmental conditions on social systems. There is an intricate and complex connection with the cultural aspects of society that is often forgotten, but can significantly impact how ecosystems are managed (Vasseur et al. 2002a). This increased level of complexity continues to challenge the utility of the standard model of centralized governance (Hajer and Wagenaar 2003; Huiteman et al. 2009) due to a reliance on the notions of static environmental models and their predictability. This is where the subsidiarity principle also acknowledges how government can play a crucial role in finding solutions so that bottom-up approaches not only meet top-down systems but also trigger a dialogue to deal with market pressure and competition, especially coming from large corporations. This is where ecosystem governance, integrating the subsidiarity principle and SES approach, leads to not only look at the market-based framework of development but also at the protection of the ecosystem for long-term betterment of these communities. Discussions, for example, among large corporations and governments are required so as to emphasize the potential for empowerment of local people and their capacity to act. Considering that corporation’s goal is to make profits and tend to go where environmental laws might be less stringent, the mediating role of governments becomes even more critical in the context of ecosystem governance to ensure that the environment is protected, and individuals are well equipped and can participate fully in societal decision making (Vischer 2001).

The demarcation of ecosystem governance from other types of governance and management lies in the idea that ecosystems, their services and functions, in terms of quantity and quality, must be conserved in decision-making processes and policy development. Ecosystem governance is intimately linked to adaptive governance and adaptive ecosystem management as concepts that acknowledge that social-ecological systems are in constant movement and evolution (Vasseur 2016). It also means accepting the fact that no one has the perfect solution and any action should accept that ecosystems are self-organizing living entities, and that there needs to be interconnectivity among actors (from national to local governments, NGOs, private sectors, and citizens) and ecosystems. Solutions cannot be based solely on the economic valuation of the ecosystem but should also be based on the ethics of decisions making as all solutions have consequences, desirable or undesirable, intended or unintended. One solution is to ensure collaborative ecosystem governance with regional pooling of interagency, intergovernment, private, and public collaboration where knowledge of all actors can be shared to find common solutions that would be socially acceptable (Karkkainen 2002). A multiple evidence-based approach where scientific, cultural, traditional, and ecological knowledge is shared and combined can help generate innovative solutions adapted to local conditions (Tengö et al. 2014; Plante et al. 2016).

## SDGs: Increasingly tackling environmental problems?

Although all 17 SDGs are related, in direct or indirect ways, to ecosystem management and governance, four of them—SDGs 6, 13, 14, and 15—are more directly related. They are critical to achieving the other goals, calling for a new paradigm of ecosystem governance: Goal 6 on water management specifically refers to “protecting and restoring water-related ecosystems” (Target 6.6); Goal 13 on tackling climate change refers to “strengthening resilience and adaptive capacity to climate-related hazards and natural disasters” (Target 13.1); Goal 14 on the conservation and sustainable use of oceans, seas, and marine resources refers to “sustainably managing and protecting marine and coastal ecosystems to avoid significant adverse impacts, including by strengthening their resilience, and take action for their restoration in order to achieve healthy and productive oceans” (Target 14.1); and Goal 15 on the protection, restoration, and promotion of sustainable use of terrestrial ecosystems specifically refers to the “conservation, restoration, and sustainable use of terrestrial and inland freshwater ecosystems and their services” (Target 15.1) (Fig. 1).

Under the definition of ecosystem governance and in the context of SES, if a resource is sustainably used, it should mean that it and its encompassing ecosystem are conserved and should not impact on other ecosystems (Liu et al. 2015). This notion relates to framing conservation as “people- and nature-centric” (or human-nature nexus as referred to in Liu et al. 2015), where the focus moves on a telecoupling framework, i.e., considerations of distant environmental, social, and economic interactions (sensu Liu et al. 2015), and “fully away from a focus on species and protected areas and into a shared human-nature environment, where the form, function, adaptability, and resilience provided by nature are valued most highly” (Mace 2014, p. 1559). This approach has already been successful in the case of the Miyun Reservoir watershed providing water to Beijing, China, with the Paddy Land-to-Dry Land program to reduce the impacts of rice cultivation on use of water through payment for ecosystem services (Zheng et al. 2013).

The other 13 SDGs also benefit from enhanced governance and a shared view of people and nature that should ultimately be supported by the five environment-related goals. For instance, Goals 4 and 7 can benefit in many ways as ecosystem representation and ethics become important. Goal 4 (inclusive and equitable quality education and lifelong learning opportunities) is crucial to increasing awareness and knowledge of how to improve ecosystem governance. Inclusion of these concepts in school curricula remains a challenge even in developed countries where sustainability is barely taught due to financial or time constraints, lack of knowledge or interest from teachers (Janzen 2016).

The SDGs bring a new dimension and imply that there are unavoidable wicked problems. Goal 13 (Take urgent action to combat climate change) recognizes climate change as a wicked challenge that needs to be addressed in order to ensure sustainable development. This goal is intricately linked to the UNFCCC and the Paris (and follow-up Marrakesh) Agreement and pushes for the support of all countries due to the global scale and wicked nature of the problem. Tackling climate change as a sustainable development goal demonstrates the need for ecosystem governance, and will require novel approaches based on the recognition that human social and natural systems are intricately interconnected (Cote and Nightingale 2012) and “to overcome the current challenges, one must understand how to connect top-down national policies to … bottom-up development strategies” (Vasseur and Jones 2015). It requires acting locally, and finding ways to encourage dialogue within and among government agencies, which can encourage other communities, so that each ecosystem and community will have to modify solutions in function of their needs and conditions.

There are opportunities through some of the international programs such as REDD (Martınez de Anguita et al. 2013). REDD + policies are usually developed at the national level with very little benefits for local communities. This was observed in San Juan, Chimborazo, Ecuador, where eucalyptus were planted with no understanding of local needs and environmental conditions and are now threatening local agriculture and livelihoods due to encroachment (Vasseur, pers. obs.). Using an ecosystem governance approach, it would be possible for countries to develop a national policy while at the same time leaving local stakeholders responsible for taking care of resources. Because they are part of the solutions, they can better define their needs and even develop a local market (Martınez de Anguita et al. 2013). One interesting example of such an approach (although not included in the reforestation scheme of the country) is a 10 ha forest in Cumanda, Chimborazo, Ecuador, where the owner decided about 10 years ago to attempt to grow a forest that looks more like a natural forest while still being functional, instead of the normal monoculture plantations of his neighbors. This functional forest is very diverse from the ground vegetation to trees and epiphytes and generates enough income to keep five people employed. Trees are only cut when there is a demand for a specific species (e.g., for door or furniture making). Cacao is harvested for the market as well as fruits (e.g., banana, orange). This ecosystem governance alternative has led to increased biodiversity and carbon sequestration, and restored ecosystem while employing people in the community for common good (Cerda and McLaren 2016).

SDG 13 must acknowledge that the resilience of complex adaptive SES can flourish only if ecosystem governance is integrated at the national policy level, and also through devolution of rights and responsibilities to communities, “as local communities tend to be culturally more homogenous than communities lumped at regional or national levels and more able to negotiate and resolve differences” (Vasseur and Jones 2015, see also Crona and Bodin 2006). Interestingly, such an approach (which can be considered under a telecoupling framework, Liu et al. 2015) would strengthen resilience and adaptive capacities to climate change, and may assist in achieving other goals, such as food security (Goal 2), and conservation and sustainable use of natural resources (Goals 14 and 15), and provide a framework that would be more conducive to pursuing activities that would respond to the wicked nature of many of the problems that SDGs aim to address. A recent publication from IUCN (Cohen-Shacham et al. 2016) demonstrates that through nature-based solutions, several of these actions are possible and are mainly anchored at the local level, as it was the case of the farmer in Cumanda, Ecuador (Cerda and McLaren 2016). Another initiative, “Community Adaptive Life Plans,” is underway in Colombia (Andrade pers. comm.).

This integrated approach requires governments to accept that economic growth at the national level is not a long-term viable solution (Norgaard 2010). Rather, new approaches are encouraged to refocus on elements such as safe, resilient, inclusive, and sustainable cities and human settlements (Goal 11; see Thornbush et al. 2013), promoting decent work and healthy living for all (Goals 3 and 8); promoting gender equity and education for all (Goals 4 and 5); and sustainable consumption and production (Goal 12). These all need to be supported by open and transparent decision-making processes, adaptive governance, and accountability at the lowest appropriate level. So what do these goals have in common? They are based on the premise of ecosystem governance (Karkkainen 2002). If we are to achieve the SDGs, the focus has to shift to a more integrated social-ecological platform where progress can be measured and monitored based on more comprehensive indicators, accessible and collaboratively developed with communities (Tengö et al. 2014). It may require regulatory reform to allow for greater flexibility, adaptive responses, and new scale relevant governance (Odom Greene et al. 2015). We understand that this may also lead to a major refocusing of the development agenda for many countries.

## SDGs and an alternative future

It is clear that the SDGs are intricately connected among themselves and with ecosystem governance, despite being categorized under 17 different goals. Unfortunately, the current way to categorize environment, economy, and society separately does not recognize this interconnectivity hindering effective cross-sectoral action, further contributing to the marginalization of the environmental sector (and very often its social and cultural counterparts) and its crucial role in achieving SDGs. Without a substantial re-framing of governance of the world’s ecosystems that recognizes the foundational role that ecosystems play, it is unlikely that any meaningful progress can be made towards the 17 SDGs. Some countries that have the resources may achieve some of the SDGs, but many LDCs will continue to find them difficult to achieve due to historic destruction and degradation of their ecosystems. Denying the interconnections between overexploitation of fisheries, forests or biodiversity, and poverty or human health, for example, will impede the capacity of countries to move forward on the path of sustainability (Liu et al. 2015). Sustainability is a process that requires that all aspects of the ecosystem, from services to human needs, are considered as one, thereby further making the case for embracing ecosystem governance.

National governments embracing ecosystem governance will help all classes of citizens benefit from the implementation of SDGs. The UN, international bodies, and the implementation of global agreements must accept the need to provide resources to support the emergence of new organizations and institutions based on new governance models, as explained in the case of the REDD + program (Martinez de Anguita et al. 2013). The development of national policies matched with local community participation under this program has shown potential such as the case of the Juma REDD Project in Novo Aripuana, Amazonas, Brazil, where the Bolsa Floresta PES program was implemented by the Foundation Sustainable Amazonas (http://fas-amazonas.org/pbf/). It is also equally important that cultural, ecological, and other existing knowledge be respected alongside all ecosystem services that are essential for poverty reduction, food security, health, and wellbeing. Resources are not only monetary but also material, educational, and human.

As mentioned by Martinez de Anguita et al. (2013), for countries to hold their commitments under international conventions, devolution to the lowest accountable body can support enhanced local ecosystem governance. Odom Greene et al. (2015) argue that adaptive management and the acknowledgement of SES complexity can be integrated into legal frameworks to support resilience-based governance. More supportive openness and opportunities from domestic and international funding sources are needed to support ecosystem governance at the global, national, and local levels. This is starting to happen in some countries with devolution to a lower accountable body, such as in Tanzania (Kangalawea and Noe 2012). Adaptive ecosystem governance should lead to greater transparency and inclusiveness and thus reduce the current challenges of corruption and personal economic gain that plague many governments and corporations (Martinez de Anguita et al. 2013). This is the case with the Amazonas Sustainable Foundation where transparency of financial activities of the projects and decisions are clearly promoted while partnering with over one hundred governmental and non-governmental organizations (http://fas-amazonas.org/). This example demonstrates the importance of maintaining transparency to ensure equity and efficiency in the decisions and actions carried out at the local level.

These ideas of integrating humans and ecosystems in SES and pushing for ecosystem governance to achieve the SDGs are not new. They were suggested in 2000 in the wake of the MDGs. At the Ecosummit 2000 in Halifax, Canada, for instance, participants concluded that to achieve sustainable development and protect human and ecosystem health, solutions should focus on the following:

1. Maximizing global human wellbeing;
2. Ensuring long-term ecological sustainability/integrity;
3. Preserving all aspects of biodiversity; and
4. Creating the necessary linkages/connections for sustainable development (Vasseur et al. 2002b, p. 200).

We argue in this paper that these principles can be implemented if ecosystem governance including adaptive management based on a human-nature nexus framework with an emphasis on subsidiarity can be adopted by nations where governments also encourage more sustainable policies such as low carbon economy, etc. This may require greater reflection and understanding of the role of ecosystem governance from larger international bodies such as the United Nations and WTO to lead to the realization that a more integrated cross-sectoral approach (possibly using telecoupling framework, Liu et al. 2015) with a balance in SES as one of the first priorities to achieve the SDGs.

The principles on which SDGs should acknowledge an ecosystem governance approach and where the ecosystems and services they provide are managed, restored, and governed to support the SDGs should be implemented in a manner that promotes sustainable development and human wellbeing. All such policies should be based on an understanding of the limits of ecosystems (and the planetary boundaries). Future actions should include the following:

1. Adoption of adaptive ecosystem governance approaches, where communities and decision makers connect with, and mutually respect each other, in a fully participatory process;
2. Respect for ecosystem services and biodiversity through their conservation, restoration, and sustainable adaptive management that also accept the need for monitoring and social learning;
3. A broader valuation of ecosystem services and biodiversity that goes beyond simply defining them as tradable commodities that can be subject to a financial cost-benefit analysis for decision making; and
4. Acknowledgement of the existence of “messy” or “wicked” problems (e.g., climate change, population growth, land degradation) that cannot be addressed by simple solutions will require thinking “outside the box” for solutions that do not currently exist.

Business as usual is not an option and countries will be unable to avoid the impacts of further ecosystem degradation and loss of functionality if they maintain the current economic and development systems. To achieve such ambitious principles and support the SDGs, we believe that science (natural, human), law and policy, the private sector, public actors, and governments (from local to international) must co-produce (i.e., producing solutions in a cooperative, inclusive, and participative manner) solutions through a common language and knowledge that respects existing knowledge (cultural, traditional, ecological, etc., Tengö et al. 2014) and institutional systems. We admit that this is a challenge, as many still adhere to the business as usual model as being the right approach. But without such a drastic change in approach, we seriously doubt that all SDGs will be achieved within the next 15 years, making the approval of the 2015 SDGs futile.

## Conclusion

We argue that it is critical to integrate adaptive ecosystem governance into national policies and relevant international conventions. Too many examples show that we are far from reaching sustainability if we maintain the current business as usual model. We have identified a number of major barriers that must be overcome in order to move forward with sustainable development. The integration of ecosystem services and their valuation into an integrated approach to development planning is essential to better understand the complex dynamics of ecosystems and sustainable socio-economic development. Considering the challenges to defining and developing solutions to current (not considering future) problems to be addressed by the SDGs, it is only through an acceptance of ecosystem governance model that we can find more appropriate actions and strategies to solve these problems. Strategies will have to be defined respecting cultural, historical, and ecosystem contexts. It will require a deeper commitment of all communities as well as private and public sectors.

The key to the success of the SDGs is the effective engagement of all parties, from local to global, in order to ensure that all actors can be involved using the principles of adaptive ecosystem governance. Economic growth at the national scale cannot be the sole focus for sustainable development, and does not represent the optimal long-term solution. Rather, adaptive ecosystem governance is required to achieve sustainable development. Governance devolved at new local inclusive institutions, where adaptive ecosystem management can be undertaken, may need national and international policies to support innovation and diversity of initiatives. Action plans should include strategies such as multiple evidence base and participatory action research approaches, to assess the status of the SES, and build alternative models that can promote strategies that protect ecosystem services for development, while respecting the plurality of cultures and religions in nations. At the international level, to monitor progress of all countries regarding the SDGs, long-term studies and a telecoupling framework could be incorporated into work plans of UN agencies and WTO for achieving the SDGs.

Capacity building remains an essential component to move forward in integrating and respecting ecosystems in the context of SDGs. The development of skills and competences in interdisciplinary work can lead to effective local civil society that can become globally responsible. Funding continues to be needed, especially in the LDCs where environmental degradation is frequently linked to development. This also means that funding should not be given blindly for development but should demonstrate the importance of ecosystem health.

Viable sustainability should consider all aspects of ecosystem governance that promotes integration of social and ecological processes necessary to deliver ecosystem services to meet human and environmental needs. Through a balanced SES, it may be possible to achieve the SDGs. To do so, ecosystem governance will require the adoption of flexible policy measures and local actions that can be adapted to changing conditions, increased community empowerment, and new learning.
